# Chemical Composition and Anti-Inflammatory, Cytotoxic and Antioxidant Activities of Essential Oil from Leaves of *Mentha piperita* Grown in China

**DOI:** 10.1371/journal.pone.0114767

**Published:** 2014-12-10

**Authors:** Zhenliang Sun, Huiyan Wang, Jing Wang, Lianming Zhou, Peiming Yang

**Affiliations:** 1 State Key Laboratory of New Drug & Pharmaceutical Process, Shanghai Institute of Pharmaceutical Industry, China State Institute of Pharmaceutical Industry, Shanghai, 200040, China; 2 Ji Lin Medical College, Ji Lin, 132013, China; 3 Department of General Surgery, Fengxian Hospital affiliated to Southern Medical University, Shanghai, 201499, China; Duke University Medical Center, United States of America

## Abstract

The chemical composition, anti-inflammatory, cytotoxic and antioxidant activities of essential oil from leaves of *Mentha piperita* (MEO) grown in China were investigated. Using GC-MS analysis, the chemical composition of MEO was characterized, showing that it was mainly composed of menthol, menthone and menthy acetate. MEO exhibited potent anti-inflammatory activities in a croton oil-induced mouse ear edema model. It could also effectively inhibit nitric oxide (NO) and prostaglandin E2 (PGE2) production in lipopolysaccharide (LPS)-activated RAW 264.7 macrophages. The cytotoxic effect was assessed against four human cancer cells. MEO was found to be significantly active against human lung carcinoma SPC-A1, human leukemia K562 and human gastric cancer SGC-7901 cells, with an IC_50_ value of 10.89, 16.16 and 38.76 µg/ml, respectively. In addition, MEO had moderate antioxidant activity. The results of this study may provide an experimental basis for further systematic research, rational development and clinical utilization of peppermint resources.

## Introduction

Inflammation is regarded as an important baseline reaction responsible for manifestations of various chronic diseases such as cancer, septic shock, diabetes, atherosclerosis and obesity [1]. Recent data have expanded the concept that inflammation is a critical component of tumor progression. Many cancers arise from sites of infection, chronic irritation and inflammation. It is now becoming clear that the tumor microenvironment, which is largely orchestrated by inflammatory cells, is an indispensable participant in the neoplastic process, fostering proliferation, survival and migration [2]. The search for more effective and safer anti-inflammatory and anticancer compounds has continued to be an important area of active research. Many traditional medicines, essential oils and volatile constituents extracted form aromatic plants have been widely used as anti-inflammatory, antitumor, antioxidant and antimicrobial agents for the prevention and treatment of different human diseases, such as cancer, cardiovascular diseases (including atherosclerosis and thrombosis), bacterial and viral infections [3]. Essential oils derived from several plants have been found to have anti-inflammatory, cytotoxic and antioxidant activities [4]–[6].

Peppermint (*Mentha piperita* L.) is a cultivated natural hybrid of *Mentha aquatica* and *Mentha spicata* L. Although *M. piperita* is a native genus of the Medierranean region, it has been spread all over the world for use in flavor, fragrance, medicinal and pharmaceutical applications [7]. Modern pharmacology research has indicated that the entire herb of *M. piperita* possesses antioxidant, cytotoxic, antiallergenic, antiviral and antibacterial activities [8]. The essential oil of *M. piperita* is reported to have antimicrobial and antioxidant activities [9], [10]. The widespread use of *M. piperita* in traditional medicines has inspired us to explore its potential biological activities, knowing that there are few previous studies reporting the cytotoxic and anti-inflammatory activities of the essential oil of *M. piperita* (MEO). Here we report the chemical composition of essential oil extracted from the leaves of *M. piperita* grown in China and its anti-inflammatory, cytotoxic and antioxidant activities.

## Materials and Methods

### Plant materials and chemicals

Leaves of *M. piperita* were collected from the Experimental Halophytes Growing Base of Shandong Academic of Sciences (Jinan, China) in August 2012. The Shandong Academic of Sciences is responsible for the Experimental Halophytes Growing Base. Shandong Academic of Sciences is the institution directly under the government of Shandong province. The location of this study is 117.2620 degrees east longitude and 36.6599 degrees north latitude. No specific permissions were required for this location and research. This research field did not involve endangered or protected species. Dulbecco's modified essential medium (DEME) and other cell-culture reagents including FBS fetal bovine serum (FBS) were obtained from GIBCO Inc, NY, USA. lipopolysaccharide (LPS), 3-(4,5-dimethylthiazol-2yl)-2,5-diphenyltetrazolium bromide (MTT), 1,1-diphenyl-2-picryl-hydrazyl (DPPH), Butylated hydroxytoluene (BHT) and ascorbic acid were purchased from Sigma-Aldrich (St. Louis, USA). All other chemicals and solvents used in this study were of reagent grade.

### Ethics statement

All the procedures were in strict accordance with the PR China legislation on the use and care of laboratory animals and with the guidelines established by the Institute for Experimental Animals of Shandong University, and were approved by the research ethics committee of Shandong University for animal experiments.

### Essential oil preparation

Leaves of *M. piperita* were subjected to hydrodistillation for 6 h in a Clevenger-type apparatus. The essential oil of *M. piperita* (MEO) was dried using anhydrous sodium sulphate as drying agent and stored under at 4°C in the dark for further analysis.

### GC-MS analysis

GC-MS analysis was performed on an Agilent 19091S apparatus, fitted with a fused silica HP-1 capillary column (30×0.25 cm id; 0.50 µm film thickness), and coupled to an Agilent Mass Selective Detector MSD 5973. The temperature program was increased to 100°C for 0.5 min, then increased to 280°C at a rate of 4°C/min, and maintained for 20 min. The other parameters were as follows: injection temperature, 280°C; EI, 70Ev; Carrier gas, He at 1 ml/min; injection volumn, 1 µl; split ratio, 1∶60; and mass range, m/z 40−600.

### Assay anti-inflammatory activity *in vivo*

Male ICR mice aged 4-week-old and weighing 18–22 g (Center for New Drugs Evaluation of Shandong University, Jinan, China; Certificate No. SKXK. 20030004) were acclimatized for a week before the experiment with free access to rodent laboratory chow and tap water and maintained under 24±1°C, humidity of 50±10%, and 12/12 h light/dark cycle. All the procedures were in strict accordance with the PR China legislation on the use and care of laboratory animals and with the guidelines established by the Institute for Experimental Animals of Shandong University, and were approved by the university committee for animal experiments.

To estimate the inhibitory activity of MEO, a mouse model of croton oil-induced ear edema was established according to the previous method with minor modifications [11]. Briefly, 10 µl acetone containing 5% croton oil was applied topically to the right ear of 4-week-old male ICR mice weighing 18–22 g. MEO (200, 400 and 800 µg/ear) and indomethacin (300 µg/ear) were applied topically to the right ear about 60 min before the croton oil treatment. The left ear received an equal volume of acetone. The mice were sacrificed by cervical dislocation in 4 h, and the plug (5 mm in diameter) was removed from both treated (right) and untreated (left) ears. The edematous response was measured as the weight difference between the two plugs.

### Determination of nitric oxide (NO) and prostaglandin E2 (PGE2) production

To investigate the anti-inflammatory activity of MEO, NO and PGE2 production in LPS-stimulated murine macrophage RAW 264.7 cells was examined. RAW 264.7 cells (Shanghai Institute of Cell Biology, Shanghai, China) were cultured in Dulbecco's modified Eagel's medium (DMEM, GIBCO Inc, NY, USA) supplemented with 100 U/ml penicillin, 100 µg/ml streptomycin and 10% FBS. Cells were incubated in an atmosphere of 5% CO_2_ at 37°C and subcultured every 3 days.

For NO determination, RAW 246.7 cells were seeded in 96-well plates at a density of 2×10^5^ cells/well and grown for 2 h for adherence, treated with MEO (5, 25, 50 and 100 µg/ml) for 1 h, and then incubated for additional 24 h in fresh DMEM with or without 1 µg/ml LPS. The nitrite concentration in the culture medium was measured as an indicator of NO production according to the Griess reaction [11]. Briefly, 100 µl cell culture supernatant were reacted with 100 µl Griess reagent [1∶1 mixture of 0.1% N-(1-naphthyl) ethylene-diamine dihydrochloride in water and 1% sulfanilamide in 5% phosphoric acid] in a 96-well plate, and absorbance at 540 nm was recorded using the ELISA reader.

For PGE2 determination, RAW 264.7 cells were seeded in 96-well plates at a density of 1×10^4^ cells/well and incubated for 18 h. Different concentrations of MEO were diluted with DEME before treatment. Cells were treated with LPS 1 µg/ml for 24 h to allow cytokine medium production. The PGE2 concentration in the culture medium was quantitated using a competitive enzyme immunoassay kit according to the manufacturer's instructions. The production of PGE2 was measured relative to that following control treatment. All the experiments were performed in triplicate.

### Assay for cell viability

Cell viability assay was determined on the basis of MTT assay as described previously with minor modifications [12]. After culture, supernatants were collected for NO or PGE2 measurement by adding 100 µl tetrazolium salt solutions (1 ml MTT in 10 ml DMEM) to each well, and then incubated for 1 h at 37°C in a 5% CO_2_ incubator. The medium was then aspirated, and the insoluble formazan product was dissolved in 100 µl DMSO. The extent of MTT reduction was quantitated by measuring the absorbance at 570 nm.

### Assay for cytotoxic activity

Human lung carcinoma SPC-A1 cells, human gastric cancer SGC-7901 cells, human leukemia K562 cells and human hepatocellular carcinoma BEL-7402 cells were obtained from the Cell Bank of the Chinese Academy of Sciences (Shanghai Institute of Cell Biology, Shanghai, China). SPC-A1, SGC-7901, K562 and BEL-7402 cells were grown in RPMI-1640 medium supplemented with 10% FBS, penicillin (100 U/ml) and streptomycin (100 µg/ml). Cells were maintained at 37°C in a humidified incubator with 5% CO_2_, and regularly examined using an inverted microscope. The medium was replaced every two days and cells were sub-cultured at 70%–80% confluence.

MTT colorimetric assay was used to evaluate the cytotoxic effect of MEO. SPC-A1, SGC-7901, K562 and BEL-7402 cells were placed into 96-well culture plates (5×10^3^), respectively, and allowed for 8 h. Cells were treated with different concentrations of MEO for 24 h. After addition of 150 µl PBS containing 0.5 mg/ml MTT, cells were incubated at 37°C for 4 h. Formed formazan crystals were dissolved in 150 µl DMSO. Absorbance in the control and drug-treated wells was measured at 490 nm with an ELISA reader.

### Determination of antioxidant activities

#### DPPH radical scavenging assay

DPPH radical scavenging assay was performed according to the reported method with some modifications [13]. Briefly, 0.5 ml various dilutions of MEO were added to 2 ml 0.004% methanol solution of DPPH. The mixture was shaken vigorously and tranquilized for 30 min at room temperature in darkness. Absorbance (A) was measured at 517 nm. The DPPH radical-scavenging activity was then calculated according to the following equation:

Scavenging rate (%)  =  [(A_control_−A_sample_)/A_control_×100]

Where A_control_ is the absorbance of the control reaction (containing all reagents except the samples) and A_sample_ is the absorbance in the presence of the sample. BHT was used as positive control.

### Reducing power

The reducing power was determined by the method described in the literature [13]. Different concentrations of 1 ml MEO were mixed with 2.5 ml 0.2 mol/l phosphate buffer (pH 6.6) and 2.5 ml 1% potassium ferricyanide. The resulting mixture was incubated at 50°C for 20 min. The reaction was terminated by adding 2.5 ml 10% trichloroacetic acid, and the mixture was centrifuged at 6000 *g* for 10 min. Finally, 2.5 ml upper layer was mixed up with 2.5 ml water and 0.5 ml 0.1% FeCl_3_, and the absorbance of the resulting prussian blue was measured at 700 nm. BHT was used as positive control.

### Hydroxyl radical scavenging activity

Hydroxyl radical scavenging activity was measured according to Fenton method described before [13]. Different concentrations MEO (200, 400, 600, 800 and 1000 µg/ml) were prepared, and incubated with 9.0 mM FeSO_4_ (1.0 ml), 0.3% H_2_O_2_ (1.0 ml) in 0.5 ml salicylic acid–ethanol solution (9.0 mM) for 30 min at 37°C. Hydroxyl radical was detected by monitoring absorbance at 510 nm. The total volume of the mixture in each tube was made up to 3 ml by adding the required amount of distilled water. The hydroxyl radical scavenging effect was calculated as follows:

Scavenging rate (%)  =  (A_control_ − A_sample_)/A_control_ ×100

Where A_control_ and A_sample_ represent the absorbance of the blank control group and sample group under 510 nm, respectively. BHT was used as positive control.

### Total antioxidant activity

The assay was based on the reduction of Mo (VI) to Mo (V) by the extract and subsequent formation of a green phosphate/Mo (V) complex in acid medium [14]. 0.3 ml sample solution was combined with 3 ml reagent solution (0.6 M sulfuric acid, 28 mM sodium phosphate and 4 mM ammonium molybdate). The tube was incubated at 95°C for 90 min. After cooling the mixture to room temperature, the A value of the solution was measured at 695 nm against the blank. The antioxidant activity is expressed as the number of equivalents of ascorbic acid.

### Statistical analysis

Data were expressed as means±SD. Statistical analysis was performed by using the Student's t-test. Different were considered significant at P≤0.05. The inhibitory concentration 50% (IC_50_) was calculated from the Prism dose-response curve, obtained by plotting the percentage of inhibition versus the concentration.

## Results and Discussion

### MEO chemical composition

**Figure 1:**
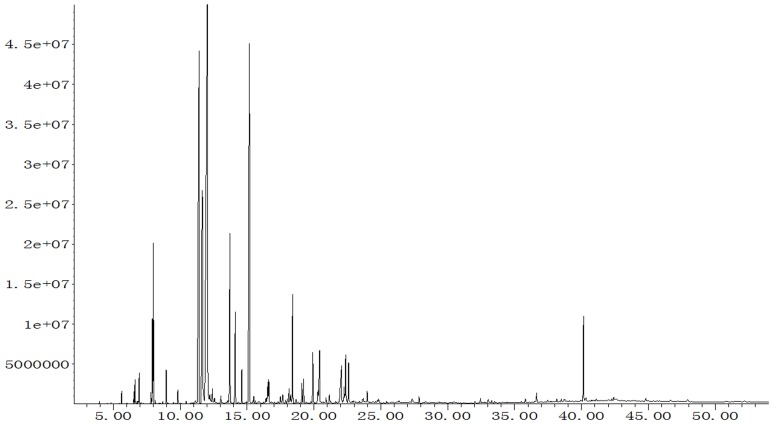
GC/MS of MEO.

In MEO chromatographic analysis (Fig. 1), 51 compounds were identified based on comparison of the mass spectrum with the NIST08 database and the related literature [15], [16], [17], representing 96.53% of the total oil (Table 1). The MEO was characterized by the dominant presence of menthol (30.69%), menthone (14.51%) and menthy acetate (12.86%), in agreement with other authors, such as Lu et al, who identified menthol, menthone and menthy acetate occurring at 32.53%, 26.50% and 10.37% in samples collected in Jiangsu province, China, Xu et al, who found them at 45.52%, 17.21% and 4.41% in samples collected in Xinjiang Province in China [15], [16]. Other significant constituents in MEO included neomenthol (9.26%), pulegone (4.36%), cineol (2.91%) and caryophyllene (2.52%). Although the contents of these main constituents in essential oil derived from *M. Piperta* grown in China were similar, there is a difference in chemical composition of MEO compared with those grown in other regions of the world. In essential oil from *M. Piperta* collected in Iran, researchers reported α-teripene as the major constituent (19.7%), followed by pipertitinone oxide (19.7%) and isomenthone (10.3%) [9]. The main constituents in MEO derived from India were reported to be limonene (18.4%), α-pinene (17.3%) and β-pinene (13.9%) [17]. However, we did not find menthofuran in MEO in the present study. This variation in chemical composition may be attributed to various factors in growing conditions, such as temperature, humidity, radiation, climate and harvest seasons.

**Table 1:** Chemical composition of MEO.

No.	R.t.^a^	KI^b^	Molecular weight	Chemical constituents	(%)
1	5.627	924	136	α-Pinene	0.20
2	6.538	964	136	Sabinene	0.20
3	6.623	969	136	β-Pinene	0.46
4	6.936	980	136	β-Myrcene	0.52
5	7.815	1011	134	Cymene	0.22
6	7.923	1018	136	D-Limonene	1.76
7	7.998	1029	154	Cineol	2.91
8	8.13	1035	136	β-trans-Ocimene	0.07
9	8.958	1047	154	Terpineol, cis-,β-	0.71
10	9.827	1082	154	Linalool	0.42
11	10.444	1130	154	2-Cyclohexen-1-ol, 1-methyl-4-(1-methylethyl)-, trans-	0.07
12	11.14	1154	154	Isopulegol	0.15
13	11.407	1166	154	Menthone	14.51
14	11.66	1173	156	Neomenthol	9.26
15	12.052	1181	156	Menthol	30.69
16	12.216	1206	156	Cyclohexanol, 5-methyl-2-(1-methylethyl)-, (1α, 2α, 5α)-	0.45
17	12.404	1214	154	Terpilenol	0.41
18	12.569	1236	148	Estragole	0.30
19	13.615	1264	138	Carane	0.07
20	13.719	1288	152	Pulegone	4.36
21	14.118	1294	152	Pipertone	2.31
22	15.175	1304	198	Menthy acetate	12.86
23	15.501	1322	198	Levomenthol	0.17
24	15.614	1335	196	Isopuegyl acetate	0.08
25	16.245	1357	204	Bicyclogermacrnene	0.06
26	16.824	1375	164	Eugenol	0.11
27	17.498	1398	204	α-Bourbonene	0.23
28	17.666	1405	204	β-Elemene	0.29
29	17.874	1414	164	Jasmone	0.09
30	18.404	1425	204	Caryophyllene	2.52
31	19.229	1440	204	β-Farnesene	0.54
32	19.782	1481	204	Naphthalene, 1,2,4a,5,6,8a-hexahydro-4,7-dimethyl-1-(1-methylethyl)-	0.06
33	19.918	1497	204	Germacrene	1.13
34	20.421	1514	204	2(4H)-Benzofuranone, 5,6,7,7a-tetrahydro-3,6-dimethyl-	1.69
35	20.903	1537	204	Cadinene	0.18
36	22.063	1587	154	β-(3-Thienyl)acrylic acid	2.09
37	22.253	1619	220	Spathalenol	0.41
38	22.372	1630	220	Caryophyllene oxide	1.37
39	22.589	1654	222	Ledol	1.05
40	22.966	1676	222	3-Hexadecyne	0.11
41	23.69	1688	222	α-Cadinol	0.16
42	23.833	1697	220	Aristolene epoxide	0.10
43	23.98	1741	222	Eupiglobulol	0.35
44	25.418	1790	236	Murolan-3,9(11)-diene-10-peroxy	0.06
45	26.345	1812	212	Benzybenzoate	0.09
46	27.345	1842	220	cis-Z-.α.-Bisabolene epoxide	0.20
47	27.857	1854	268	Hexahydrofarnesgl acetone	0.15
48	28.346	1878	238	Culmorin	0.08
49	33.03	2035	296	Phytol	0.14
50	39.956	2391	290	Grindelene	0.06
51	42.263	2404	282	Eicosanel	0.06

a Retention time (min).

b Kovats index relative to *n*-alkanes (C_9_–C_25_) on HP-1 capillary column.

### *In vivo* anti-inflammatory activity assay

**Figure 2:**
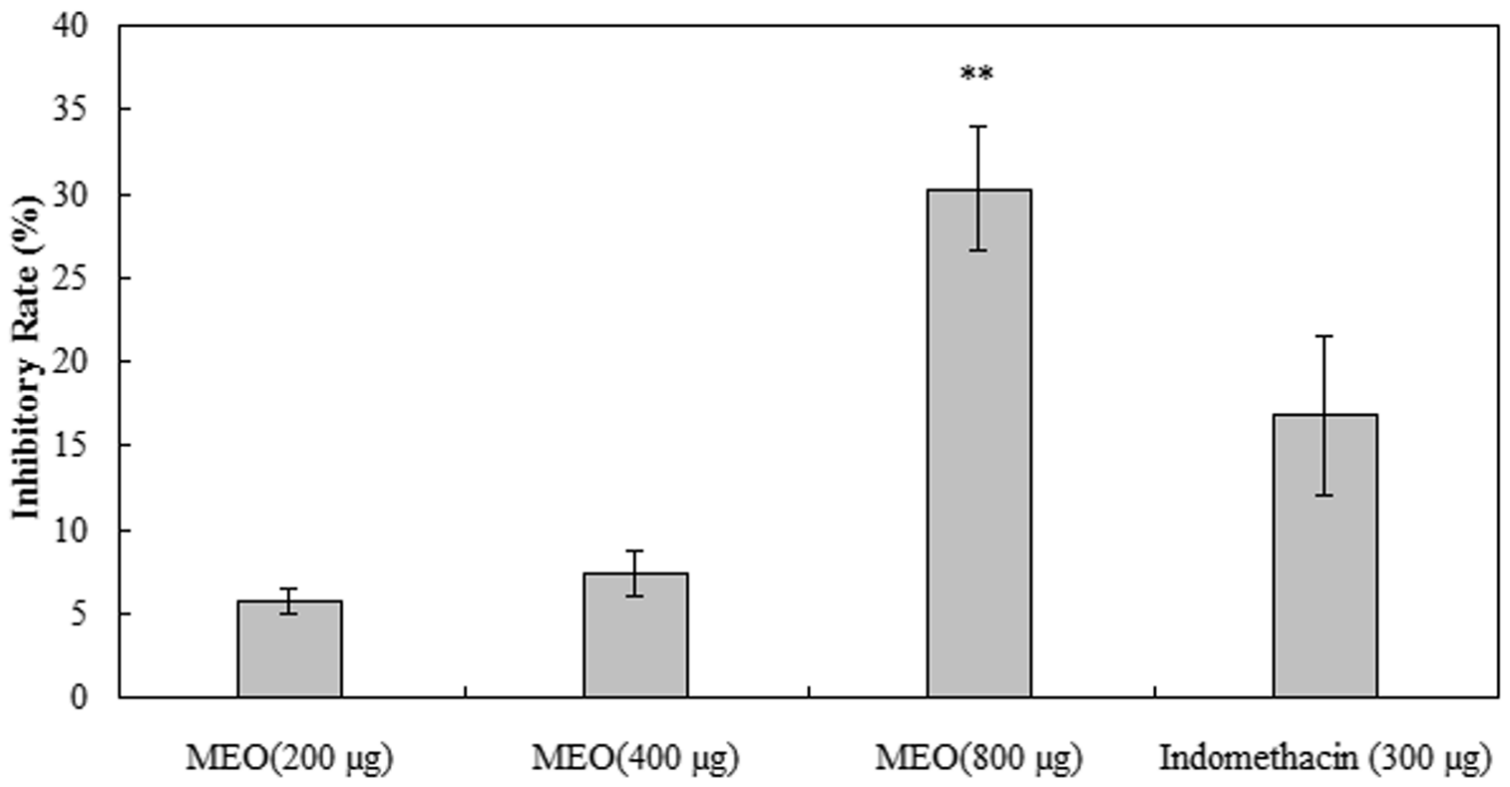
The effect of MEO against croton oil-induced ear edema in mice. Data are representative of ten experiments and as means ± SE. Indomethacin was used as a reference compound (300 µg per ear). Statistical differences from indomethacin-treated control as analyzed by Dunnett's test (** *P*<0.01)

The anti-inflammatory activity of MEO in the croton oil-induced mouse ear edema model was evaluated in this study. As shown in Fig. 2, in comparison with the non-steroidal anti-inflammatory drug indomethacin, MEO exhibited a significantly high anti-inflammatory activity in a dose-dependent manner. In addition, MEO reduced the edematous response by 5.77%, 7.37% and 30.24% at the dose of 200, 400 and 800 µg, respectively. The mouse ear plug weight was reduced by 16.79% after indomethacin treatment (dose 300 µg per ear).

### Effect of MEO on NO and PGE2 production in LPS-stimulated RAW264.7 cells

**Figure 3:**
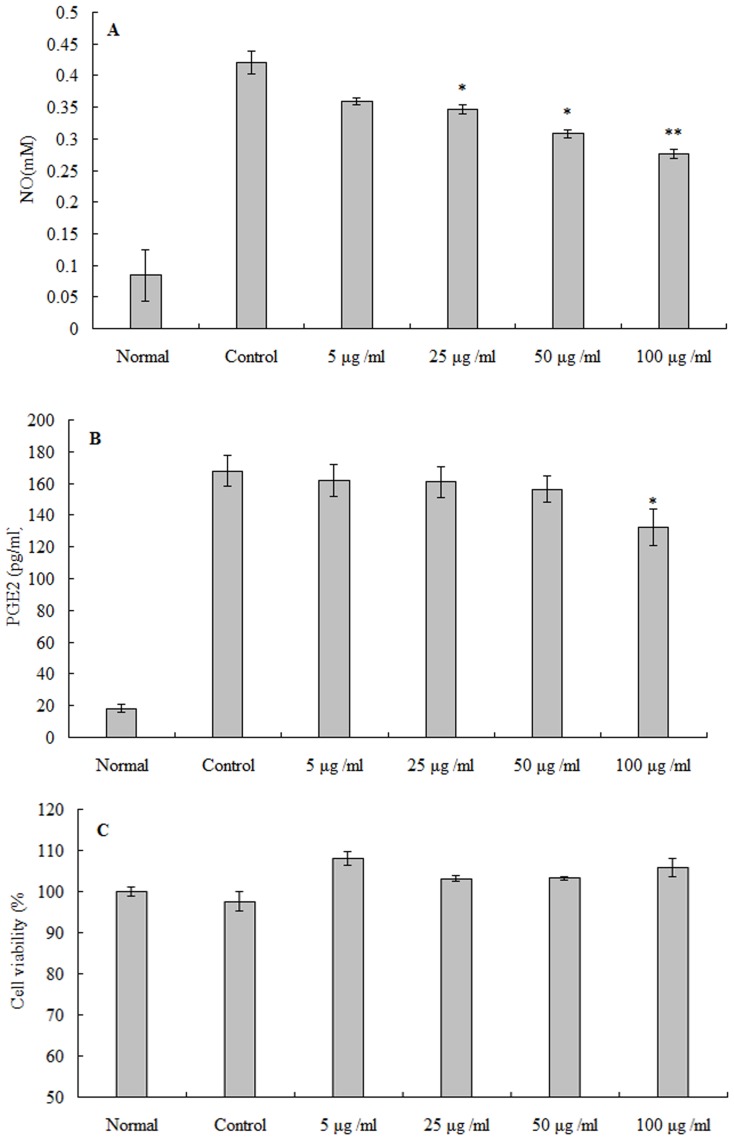
The effect of MEO on NO and PGE2 production of LPS-stimulated RAW 264.7 macrophage cells. (A) represents the inhibitory effect of NO production, (B) represents the inhibitory effect of MEO on PGE2 production; and (C) indicates the cytotoxic effect of MEO on RAW 264.7 cells. Results are mean ± SD (n = 3).

NO production was examined in RAW 264.7 cells stimulated with LPS in the presence or absence of MEO. Data in Fig. 3A showed that nitrite level increased significantly compared with that in normal cells, and MEO had a potent inhibitory effect on NO production. At the dose of 100 µg/ml, its inhibitory rate was 34.30%.

As shown in Fig. 3B, the accumulation of PGE2 in RAW 264.7 cells increased from 18.26 pg/ml to 167.90 pg/ml after induction of LPS. However, MEO showed a moderate inhibitory effect on PGE2 production. At the concentration of 100 µg/ml, PEG2 production decreased by 40%. Meanwhile, Fig. 3C showed that the number of viable activated cells remained unchanged significantly, indicating that the inhibitory effect of MEO on NO and PGE2 was not simply due to the cytotoxic effect, and MEO had no adverse effect on RAW 264.7 cells.

NO is an endogenous free radical species synthesized from L-arginine by nitric oxide synthase in various animal cells, and is recognized as a mediator in pathological reactions, especially in acute inflammatory responses [18]. PGE2 is also an important inflammatory mediator involved in the pathogenisis. Thus NO and PGE2 production is usually measured for assessing the anti-inflammatory effect. Pharmacological studies have shown that essential oil derived from various plant materials possesses anti-inflammatory activities [19], [20]. In the present study, MEO showed a notable *in vivo* and *in vitro* anti-inflammatory effect. The possible action mechanism underlying this effect may be attributed to the inhibitory effect on NO and PGE2 production. Knowing that sesquiterpenes have excellent anti-inflammatory activities [13], [20], the anti-inflammatory activity of MEO could be partly explained by the presence of sesquiterpenes, such as spathulenol, cadinene, caryophyllene and caryophyllene oxide.

### Effects of MEO on cytotoxicity of four human tumor cells

*In vitro* cytotoxic properties of MEO was evaluated in four human cancer cells (human lung carcinoma SPC-A1, human gastric cancer SGC-7901, human leukemia K562 and human hepatocellular carcinoma BEL-7402) using MTT assay. As shown in Table 2, MEO had selective cytotoxicity on different tumor cells, and a potent antiproliferative effect on SPC-A-1 (10.89 µg/ml). This potent *in vitro* antitumor effect was also shown in K562 and SGC-7901 cell assays, the IC_50_ being 16.16 µg/ml and 38.76 µg/ml, respectively. In contrast, MEO was inactive against BEL-7402 cell at the tested concentrations.

**Table 2:** Cytotoxic activities of MEO against four human tumor cell lines.

Essential oil	IC_50_ (µg/ml)			
MEO	SPC-A1	SGC-7901	K562	BEL-7402
	10.89±1.07	38.79±4.82	16.16±2.11	>100

As we did not evaluate the cytotoxic effect of all chemicals present in MEO against the four cancer cell lines, it is not possible to identify which of these compounds are responsible for the observed results. In fact, the cytotoxic effect of sesquiterpenes has been reported in the literature. Interestingly, caryophyllene was reported to exhibit anti-proliferative activity against K562 cells [21], and caryophyllene oxide inhibited growth and induced apoptosis through ROS-mediated MAPKs activation [21]. β-elemene is a broad-spectrum antitumor agent that can enhance the cytotoxic effect of radiation *in vitro* and *in vivo*, as shown by several studies [22], [23]. In addition, it can also induce apoptosis in A549 cells [24], and may represent a promising agent for overcoming MDR in cancer therapy [25]. α-cadinol was also reported to have selective toxicity against human colon adenocarcinoma cell line HT-29 [26]. In summary, the cytotoxic activity of MEO might be due to the synergic effects of different terpenes in the oil, or perhaps there are some other active compounds responsible for the cytotoxic activity of the essential oil, which deserves attention in the future.

### Antioxidant activities

#### DPPH radical scavenging assay

**Figure 4:**
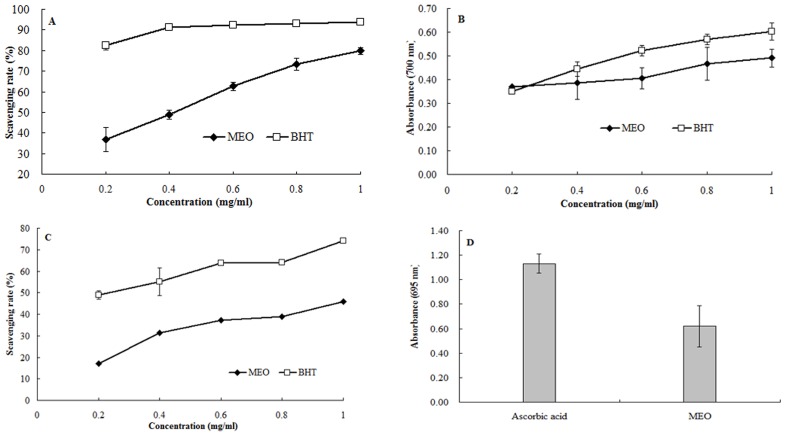
Antioxidant activities of MEO, (A) scavenging of DPPH radical, (B) scavenging of hydroxyl radical, (C) reducing power and (D) total antioxidant activity. Values were representative of three separated experiments.

DPPH is a stable free radical and has been widely accepted as a tool for estimating free radical scavenging activities of antioxidants. As shown in Fig. 4A, solutions with MEO concentrations of 200, 400, 600, 800 and 1000 µg/ml were prepared to evaluate the DPPH radical scavenging capacity, and BHT was used as positive control. The respective scavenging capacities ranged from 36.81% to 79.85% for MEO, *vs*. 82.36% to 93.85% for BHT. The scavenging ability of MEO was observed in a dose-dependent manner. Although the scavenging capacity of MEO was lower than that of BHT at all tested concentrations, the scavenging rate reached almost 80% at 1000 µg/ml, indicating that MEO had an appreciable scavenging ability.

### Reducing power

The reducing property is generally associated with the presence of reductants. The antioxidant action of reductants is based on the breaking of free radical chain by donation of a hydrogen atom. Fig. 4B shows the reducing power of MEO as compared with BHT. MEO exhibited absorbance ranging from 0.37 to 0.49, *vs*. 0.35 to 0.60 for BHT. These results indicate that MEO possesses a notable reducing power.

### Hydroxyl radical scavenging activity

Hydroxyl radical is an extremely reactive free radical formed in biological systems and has been implicated as a highly damaging species in free radical pathology, capable of damaging almost all molecules found in living cells [27]. The results concerning hydroxyl radical scavenging activity of MEO and BHT are presented in Fig. 4C. The scavenging ability of MEO was investigated at the concentration range of 0.2 to 1.0 mg/ml, and was found to be dose dependent. Although scavenging activity of BHT was lower than that of BHT, its scavenging rate reached almost 50% at the concentration of 1 mg/ml, indicating that MEO has moderate hydroxyl radical scavenging ability.

### Total antioxidant activity

Total antioxidant activity assay is based on the reduction of Mo (VI) to Mo (V) and subsequent formation of a green phosphate/Mo (V) complex in acid medium. As shown in Fig. 4D, total antioxidant capacity of MEO was expressed as the number of equivalents of ascorbic acid. MEO exhibited an effective antioxidant activity equivalent to 55% of the antioxidant activity of ascorbic acid.

Ample evidence in recent years has shown that free radical species and NO or their derivatives are the key denominators in carcinogenesis. Oxygen radicals and nitrogen oxide derivatives can effectively damage DNA causing mutations. They are probably involved in multiple steps of carcinogenesis *in vivo* [28]. In the present study, MEO showed appreciable scavenging activities on DPPH and hydroxyl radicals, notable reducing power and total antioxidant activity. The moderate antioxidant activity of MEO might be an effective action mechanism contributing to its anti-inflammatory and cytotoxic effects.

## Conclusion

In conclusion, we have identified 51 volatile constituents in MEO and evaluated its anti-inflammatory, cytotoxic and antioxidant activities. The results showed that MEO had a potent anti-inflammatory activity in the croton oil-induced mouse ear edema model, and the possible action mechanism might be attributed to its inhibitory effect on the production of NO and PGE2. MEO was also found to be active against SPC-A-1, K562 and SGC-7901 cancer cell lines. In addition, MEO had a moderate antioxidant activity. These results may serve as valuable research references for clinical research of medicines for treatment of inflammation and cancer in the future.
